# Visceral disseminated varicella zoster infection: a rare cause of acute abdomen in a patient with well-controlled diabetes mellitus—a case report

**DOI:** 10.1186/s12879-022-07183-y

**Published:** 2022-03-03

**Authors:** Daisuke Mizu, Haruka Nishida, Yoshinori Matsuoka, Koichi Ariyoshi

**Affiliations:** Department of Emergency Medicine, Kobe City Medical Center General Hospital, 2-1-1, Minatojimaminami-machi, Chuo-ku, Kobe-shi, Hyogo, 650-0047 Japan

**Keywords:** Acute abdomen, Case report, Computed tomography, Diabetes mellitus, Disseminated varicella zoster infection, Periarterial fat stranding, Varicella zoster virus

## Abstract

**Background:**

Visceral disseminated varicella zoster virus (VZV) infections frequently affect immunocompromised patients. Diabetes mellitus has been associated with VZV infection, and most cases of disseminated infection involve patients with poorly controlled blood glucose levels. It initially presents as severe abdominal pain, which is evaluated as an acute abdomen, however, the cause is typically unidentified due to unremarkable computed tomography (CT) findings. We report a case of visceral disseminated VZV infection in a patient with well-controlled diabetes mellitus with fat stranding around the celiac and superior mesenteric artery on CT.

**Case presentation:**

A 61-year-old Japanese woman with well-controlled diabetes mellitus presented to the emergency department with severe abdominal pain that gradually worsened. She had stable vital signs and skin rashes, suggestive of varicella. Abdominal CT showed fat stranding around the celiac and superior mesenteric arteries. The patient tested positive for the VZV antigen and was diagnosed with a visceral disseminated VZV infection. Acyclovir was administered, and the patient was discharged on the 14th day.

**Conclusions:**

Visceral disseminated VZV infection may affect patients with well-controlled diabetes mellitus and causes acute abdomen. Periarterial fat stranding on CT is associated with abdominal pain due to visceral disseminated VZV infection.

## Background

Varicella zoster virus (VZV) infections have a severe clinical course in immunocompromised patients. Specifically, patients who underwent bone marrow transplantation have the highest risk of developing disseminated infection [1]. Abdominal pain is often the initial symptom of visceral disseminated VZV infection, and the skin rash, characteristic of VZV infection, appears after than abdominal pain [2, 3]. Therefore, this disease is difficult to diagnosis early. However, delayed treatment can lead to multiorgan failure [4], and misdiagnosis in the emergency department negatively affects patient prognosis. In immunocompromised patients, visceral disseminated VZV infection should be considered a differential diagnosis for acute abdomen due to the high risk of developing the disease. Diabetes mellitus (DM) is also associated with VZV infection, and most cases of disseminated VZV infection involve patients with poorly controlled blood glucose levels [5, 6]. Visceral disseminated VZV infection rarely affects patients with well-controlled DM.

The cause of severe abdominal pain, associated with visceral disseminated VZV infection, remains unclear with negative imaging findings [7, 8]. Recently, periarterial fat stranding around the celiac artery (CA) and superior mesenteric artery (SMA) was reportedly confirmed by computed tomography (CT) [8–10].

We report a case of visceral disseminated VZV infection in a patient with well-controlled DM with severe abdominal pain and periarterial fat stranding on CT.

## Case presentation

A 61-year-old Japanese woman with postoperative ovarian cancer and DM, controlled with oral antidiabetic medications or drugs, presented to the emergency department (ED) with a complaint of abdominal pain that worsened over four days. She underwent surgery for ovarian cancer 11 years ago and received no chemotherapy as the disease was still at an early stage. She had been followed up as an outpatient for approximately six years and was confirmed to have no recurrence. She had other medical histories of hypertension and hyperlipidemia, with no recent history of taking any steroids or immunosuppressive medications.

**Fig. 1:**
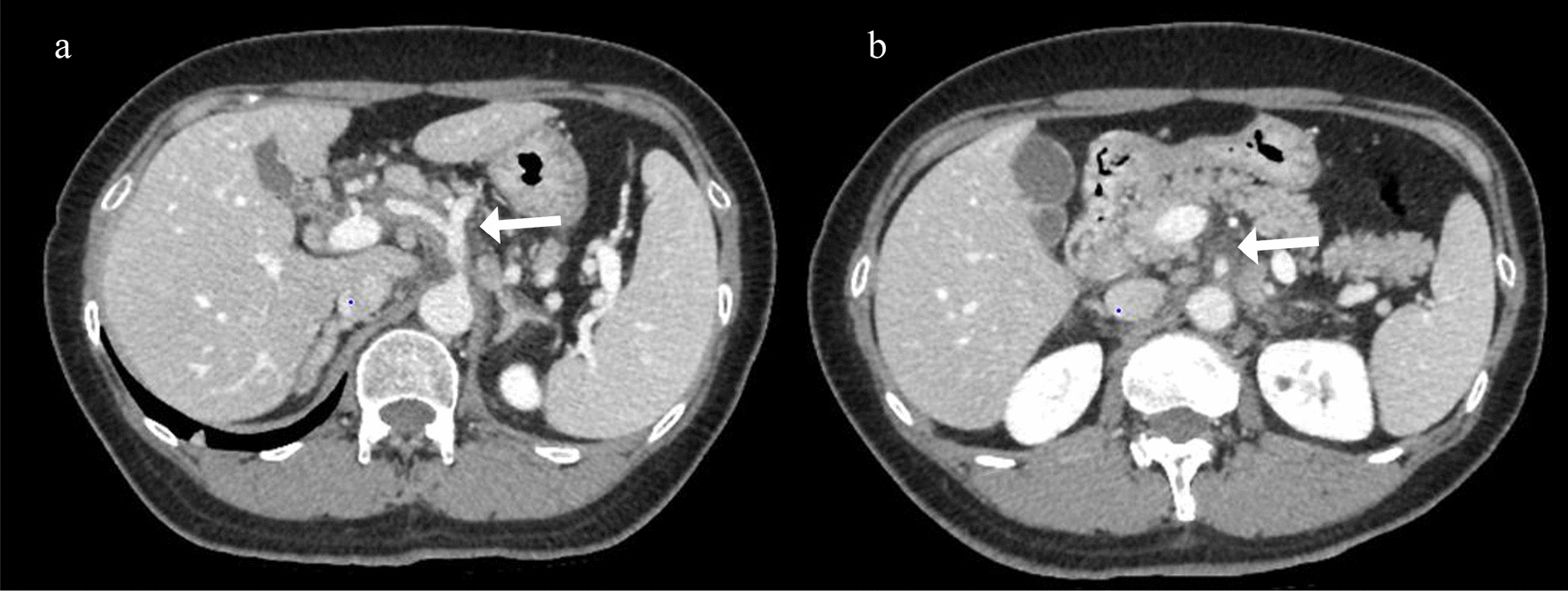
Abdominal computed tomography (CT) scan at the time of emergency department visit; **a** Increased CT value (fat stranding) around the celiac artery and its branches. **b** Fat stranding around the superior mesenteric artery

On the day of her visit to the ED, skin rashes were noted on her face, abdomen, and thighs. Within the four days between the onset of abdominal pain and the patient’s visit to the ED, she had experienced few episodes of vomiting. Abdominal examination revealed tenderness in the epigastric area without muscular guarding or rebound tenderness. The skin rashes were accompanied by blisters. The patient had a Glasgow Coma Scale of 15 (E4V5M6), blood pressure of 139/81 mmHg, heart rate of 76/min, respiratory rate of 16/min, body temperature of 35.9 ℃, and oxygen saturation level of 100% (room air). Blood tests in the ED showed a slight increase in the C-reactive protein level, as shown in Table 1. Abdominal contrast-enhanced CT, which was performed to evaluate the abdominal pain, showed periarterial fat stranding around the CA and SMA (Fig. 1a, b). No other abnormal findings were observed, and the patient tested positive for the VZV antigen and was diagnosed with visceral disseminated VZV infection. Moreover, she had severe abdominal pain, requiring fentanyl administration. Similarly, she also received acyclovir (10 mg/kg/day).

**Table 1:** Laboratory data at the emergency department

Biochemistry	Value	Normal range	CBC / coagulopathy	Value	Normal range
TP, g/dL	8	6.5–5.8	WBC, × 10^3^/μL	7.8	3.9–9.8
Alb, g/dL	4.2	3.9–4.9	Neu, %	74.5	30–70
TB, mg/dL	0.6	0.2–1.2	Lymph, %	18.5	19–61
AST, IU/L	24	8–40	Mono, %	6.5	2–12
ALT, IU/L	30	8–40	Eos, %	0.5	0–8
ALP, IU/L	182	100–340	Baso, %	0	0–2
LDH, IU/L	226	124–222	RBC, × 10^4^/μL	484	350–510
Amy, IU/L	48	40–135	Hb, g/dL	14.8	11.1–15.1
BUN, mg/dL	17.1	8–20	Hct, %	41.9	33.5–45.1
Cr, mg/dL	0.62	0.4–0.8	Plt, × 10^4^/μL	16.6	13–37
CK, IU/L	42	50–170	PT, %	103,4	80–125
Na, mEq/L	137	136–148	PT-INR	0.98	
K, mEq/L	3.3	3.5–5.3	APTT, %	100.6	60–110
Ca, mg/dl	9.5	8–10			
Glu, mg/dl	138	70–110			
CRP, mg/dl	2.13	0–0.5			
HbA1c, %	6.2				

*CBC* complete blood count, *VZV* varicella zoster virus, *TP* total protein, *Alb* albumin, *TB* total bilirubin, *AST* aspartic aminotransferase, *ALT* alanine aminotransferase, *ALP* alkalin phosphatase, *LDH* lactate dehydrogenase, *Amy* amylase, *BUN*, blood urea nitrogen, *Cr* creatinine, *CK* creatine kinase, *Na* sodium, *K* potassium, *Ca* calcium, *Glu* glucose, *CRP* C-reactive protein, *HbA1c* hemoglobin A1c, *WBC* white blood cell, *Neu* neutrophil, *Lymph* lymphocyte, *Mono* monocyte, *Eo* eosinophil, *Baso* basophil, *RBC* red blood cell, *Hb* hemoglobin, *Hct* hamatocrit, *Plt* platelet, *PT*:prothorombin time, *PT-INR* prothorombin time-international normalized ratio, *APTT* activated partial thromboplastin time

Blood tests after admission showed that both VZV-immunoglobulin (Ig)M and VZV-IgG were positive;similarly, blood VZV DNA was elevated. There was an increase in antinuclear antibodies; however, other specific antibody tests were negative (Table 2). Her abdominal pain gradually improved, and on day 5, she no longer required fentanyl. Her vital signs or blood tests did not deteriorate, and her abdominal pain was alleviated by oral acetaminophen. The patient was discharged on day 14.

**Table 2:** VZV and immunological test

VZV / immunological test	Value	Normal range
VZV-IgM/EIA	2.33	0–0.79
VZV-IgG/EIA	≥ 128	0–1.9
blood VZV-DNA load, Copy/mL	710	0–99
IgG, mg/dL	1646	870–1700
IgA, mg/dL	427	110–410
IgM, mg/dL	91	35–220
C3, mg/dL	138	65–135
C4, mg/dL	34	13–35
Antinuclear Ab	80	0–39
Anti-RNP-Ab	(−)	
Anti-Sm-Ab	(−)	
Anti-SSA-Ab	(−)	
Anti-Scl 70-Ab	(−)	
Anti-dsDNA-Ab	(−)	
MPO-ANCA	(−)	
PR3-ANCA	(−)	

*VZV* varicella zoster virus, enzyme immunoassay, *IgM* immunoglobulin M, *IgG* immunoglobulin G, *IgA* immunoglobulin A, *EIA* enzyme immunoassay, *Ab* antibody, *RNP* ribonucleoprotein, *Sm* Smith, *SSA* Sjogren syndrome A, *Scl* scleroderma, *dsDNA* double-stranded DNA, *MPO-ANCA* myeloperoxidase-anti-neutrophil cytoplasmic antibody, *PR3-ANCA* proteinase-3-anti-neutrophil cytoplasmic antibody

## Discussion and conclusions

This case highlighted two important clinical learning points. First, visceral disseminated VZV infection affected patients with well-controlled DM. Second, CT findings of periarterial fat stranding around the CA and SMA were associated with abdominal pain in visceral disseminated VZV infection.

Visceral disseminated VZV infection has commonly been reported among immunocompromised patients, such as bone marrow or renal transplant recipients, and patients under immunosuppressive therapy [1, 8–10]. In this case, the patient had a medical history of ovarian cancer, however, the surgery was performed more than 10 years ago, and no chemotherapy was administered. Therefore, the association with ovarian cancer was considered unlikely.

Additionally, disseminated VZV infection has been reported among patients with poorly controlled DM (glycated hemoglobin > 10%) [6, 11]. Previous studies have identified both type 1 and type 2 DM as risk factors for herpes zoster infection, and patients with DM had a significantly lower cell-mediated immunity to VZV [5, 12, 13]. In this case, the patient had a glycated hemoglobin of 6.2%, which indicated good control of blood glucose levels in the last one to two months. Thus, patients with DM can develop disseminated VZV infection even during periods when the blood glucose level is well-controlled. However, visceral disseminated VZV infections have not previously been reported among patients with well-controlled DM without other immunocompromising conditions, such as in this case.

Visceral disseminated VZV infection causes severe abdominal pain in 80–100% of cases [7]. VZV infection involves afferent sympathetic fibers to abdominal organs that originate from the posterior nerve roots, including cutaneous sensory fibers [14]. Disseminated VZV infection may cause inflammation of abdominal organs, such as the liver, pancreas, and intestines, due to latency and activation of the celiac ganglia, which may lead to frequent abdominal pain. Although, various etiologies of abdominal pain have been described, these remain unclear [7, 8]. Despite the appearance of severe abdominal pain, abdominal CT usually shows no specific findings in most cases. Nevertheless, there have been reports of increased fat density around the CA and SMA, as in our case [8–10, 15]. Although several conditions present with findings of periarterial fat stranding, it is unusual for fat stranding around the CA and SMA to be the isolated finding [15]. Since VZV infections have been associated with vascular disorders, especially in the cerebrovascular system [16], VZV infections may also affect visceral arteries and cause fat stranding around the CA and SMA. Furthermore, the association with visceral arteries may be a factor causing abdominal pain. Based on previous reports [8–10, 15], the isolated finding of fat stranding around the CA and SMA should be considered a probable cause of disseminated VZV infection.

In 20–60% of visceral disseminated VZV infections, abdominal pain appeared before the skin rash [3, 9]. It is difficult to diagnose this disease based on a general abdominal examination. As in this case, visceral disseminated VZV infection should be considered a cause of acute abdomen in patients with a history of DM or fat stranding around the CA or SMA on abdominal CT.

In conclusion, visceral disseminated VZV infections may affect patients with well-controlled DM. CT findings of fat stranding around the CA and SMA suggest visceral disseminated VZV infection as the cause of abdominal pain. Nonetheless, further cases are needed to confirm these findings.

## Abbreviations

CT: Computed tomography
CA: Celiac artery
DM: Diabetes mellitus
ED: Emergency Department
Ig: Immunoglobulin
SMA: Superior mesenteric artery
VZV: Varicella zoster virus
